# Protective effect of *Chrysanthemum morifolium* Ramat. ethanol extract on lipopolysaccharide induced acute lung injury in mice

**DOI:** 10.1186/s12906-020-03017-z

**Published:** 2020-07-25

**Authors:** Gang Liu, Qingxiu Zheng, Kunlei Pan, Xiaoxiao Xu

**Affiliations:** Respiratory Medicine, Wenzhou Chinese Hospital Affiliated to Zhejiang University of Traditional Chinese Medicine, No. 9 Jiaowei Road, Wenzhou, 325000 China

**Keywords:** *Chrysanthemum morifolium* Ramat. ethanol extract, Lipopolysaccharide, Acute lung injury, Total triterpenoids, Total flavonoids

## Abstract

**Background:**

To evaluate the effect of *Chrysanthemum morifolium* Ramat. ethanol extract (CEE) on lipopolysaccharide induced acute lung injury in mice.

**Methods:**

The ninety C57BL/6 J male mice randomly divided into five groups: control, model and CEE (50, 100, 200 mg/kg) groups for 7 days oral administration. At the last administration, all mice except control were intratracheal instilled with lipopolysaccharide (LPS, 3 mg/kg) for establish the acute lung injury. Then lung histopathologic, lung wet/dry weight, white blood cells, lymphocytes, neutrophils were detected. The pro-inflammation cytokine tumor necrosis factor-*α* (TNF-*α*), interleukin (IL)-6, anti-inflammatory cytokine transforming growth factor-*β*1 (TGF-*β*1), IL-10 and the marker of antioxides ability total-antioxidant capacity (T-AOC), malondialdehyde (MDA) in lung tissue were measured.

**Results:**

The result showed that CEE could improve lung histopathological injury, reduce the ratio of wet/dry lung weight and lung index, inhibit the increased number of white blood cells, lymphocytes and neutrophils, and reduce the increased levels of TNF-*α* and IL-6. While CEE also significantly increased the levels of TGF-*β*1 and IL-10. Furthermore, CEE also markedly increased the activity of T-AOC, and decreased the contents of MDA with a dose-dependent manner.

**Conclusions:**

The study exhibited that CEE has a potential protective effect on lipopolysaccharide induced acute lung injury in mice, the action mechanism of CEE may through balance of the pro-inflammatory and anti-inflammatory factors, and the oxygen free radicals inhibition.

## Background

Acute lung injury (ALI) refers to acute and progressive hypoxic respiratory failure caused by various external and internal pathogenic factors besides cardiogenic factors [1]. It is characterized by acute progressive dyspnea, decreased lung volume, decreased lung compliance, severe imbalance of ventilation/blood flow ratio, extensive injury of alveolar capillary endothelial cells and alveolar epithelial cells [2]. In 1992, the American Thoracic Society (ATS) and the European Society of Critical Diseases (ESICM) changed ARDS (adult respiratory distress syndrome) to acute, and divided it into two parts: ALI and ARDS [3]. At the same time, the causes of ALI/ARDS were classified into two major factors: direct lung injury and indirect lung injury [4–6]. A large number of clinical studies have shown that Gram-negative bacilli infection is the most common cause of ALI induction and is one of the main causes of death [7, 8]. ALI/ARDS has become a common critical disease in clinic. However, the pathogenesis of ALI/ARDS has not been fully elucidated [9]. Comprehensive treatment is the main method of clinical treatment, and there is no specific method [10, 11]. Therefore, it is of great practical significance to study the prevention and treatment of ALI/ARDS.

*Chrysanthemum morifolium* Ramat. is a dry capitate inflorescence, belongs to Compositae [12, 13]. *Chrysanthemum morifolium* Ramat. was first recorded in “Shen Nong’s Herbal Classic” and listed as the top grade, which means it is an effective, safe, non-toxic and harmless medicine [14–16]. Li et al. reported that the anti-inflammatory and antioxidant properties of CEE in vitro and in vivo [17–19]. It is often used in the clinical treatment of sores, dementia, eye swelling, lung pain, sore throat and flu. Modern pharmacological studies have shown that *Chrysanthemum morifolium* Ramat. has many pharmacological activities, such as anti-bacterial, anti-virus, anti-inflammatory, anti-oxidation, anti-cancer, treatment of coronary heart disease, lowering blood pressure, prevention of hyperlipidemia, delaying aging and so on [20].

In addition, *Chrysanthemum morifolium* Ramat. has many active ingredients, such as triterpenoids, flavonoids, volatile oils, organic phenolic acids and so on. It belongs to the antipyretic and detoxicating drugs of traditional medicine [21]. It can be used for sores and poisons, eye and face swelling, lung pain, sore throat and influenza in clinic [22]. Professor Toshihiro Akihisa, a Japanese scholar, has found that a series of triterpenoids, such as dandelion sterols from the non-saponifiable fat-soluble fraction (NSL) of *Chrysanthemum morifolium* Ramat. ethanol extract (CEE), which possess a significant anti-inflammatory activity [23, 24].

Based on the above, *Chrysanthemum morifolium* Ramat. has a wide pharmacological functions. However, to the best of our knowledge, there have not any research for the effect of CEE on lipopolysaccharide (LPS)-induced ALI in experimental mice. Thus, our study aimed to explore the effect of CEE on LPS-induced ALI in mice, and to investigate whether CEE has a certain medicinal value. The work can provide some experimental data and the theoretical basis for the development of new resources of *Chrysanthemum morifolium* Ramat. in both clinical medicine and life food in the future.

## Methods

### Materials and reagents

Malondialdehyde (MDA, Batch No 20180113) and Total antioxidant capacity (T-AOC, Batch No 20180113) purchased from Nanjing Jiancheng Biotechnology Co., Ltd. (Nanjing, China). Tumor necrosis factor-alpha (TNF-*α*, Batch No 978941030), interleukin-6 (IL-6, Batch No 1321213119), Transforming growth factor-beta 1 (TGF-*β*_1_, Batch No 9711891103) and interleukin-10 (IL-10, Batch No 1321213121) were purchased from Wuhan Boster Biology Technology co. ltd. (Wuhan, China). LPS obtained from Sigma-Aldrich (Eseherichia coli 055:B5, 818E034, USA).

### Preparation of CEE

The dried *Chrysanthemum morifolium* Ramat. was purchased from Daguan *Chrysanthemum* Tea Planting Base in Tongxu County, Qiaofeng City at October 2017, which was identified by Yanli Zhao, an associate researcher of Qiaofeng Agricultural Research Institute. The dried *Chrysanthemum morifolium* Ramat. was extracted by reflux for 2 h with 150 g and 20 times 95% ethanol, then filtered. The residue was extracted by 18 times 95% ethanol, and reflux for 3 times, 1.5 h each time, and combining the three filtrate. Then 95% ethanol was recovered by decompression and concentrated into extract, which had no alcoholic flavor and was dispersed with appropriate hot water. The extract was extracted with trichloromethane to be colorless and concentrated to extract.

The clinical daily dosage of *Chrysanthemum morifolium* Ramat. on adults (60 kg) was 5–10 g. According to the extraction rate of 95% ethanol (12.31%), the equivalent amount of the extract per gram was calculated. Then use the equivalent dose coefficient conversion algorithm to calculate the dosage of mice. The extract was prepared with 0.5% Cluster Methylcellulose Sodium (CMC-Na) solution and stored at 4 °C for use.

### Bioactive constitutes analysis by LC/MS

#### HPLC analysis

The components of CEE were separated on a Nucleodur C18 (4.6 mm × 250 mm, 5 μm) at 35 °C with the flow rate of 1.0 mL/min, the detection wavelength was 334 nm. The injection volume was 10 μL. Mobile phase was include acetonitrile (A) and 0.1% formic acid-water (B). The gradient program was: 0–5 min, 5–18% A; 5–30 min, 18–21% A; 30–32 min, 21–38% A; 32–42 min, 38–45% A; 42–45 min, 45–60% A; 45–45.01 min, 60%A-80%A; 45.01–50 min, 80%A-85%A.

#### Mass spectrum analysis

The desolvation gas was used nitrogen. The flow rate of the cone gas was 10 L/h. Capillary voltage was 3 kV for ESI (+) and ESI (−). The temperature of the ion source was 350 °C. Mass spectra were recorded in the range of m/z 50–2000 for full scan.

### Animals

Ninety C57BL/6 J male mice (body weight 20 ± 2 g, 8 weeks) were obtained from the animal experimental center of the Zhejiang University of Traditional Chinese Medicine (China), and the whole experiment was also carried out on Zhejiang University of Traditional Chinese Medicine. The mice were raised at SPF grade environment with 24 ± 1 °C, 50% ± 5% of humidity and 12 h day/night cycle. Six mice raised in one polyacrylic cage with free access to food and water. The mice were received humane care in the terms of National Institutes of Health Guidelines of the USA (National Research Council of USA, 1996) and the University ethical regulations of Zhejiang University of Traditional Chinese Medicine.

### Experimental design

The animal study protocol (Protocol number: GL-1801) approved by the animal care and use committee of Zhejiang University of Traditional Chinese Medicine. Ninety C57BL/6 J male mice were randomly divided into 5 groups: the control group, LPS model group, CEE 50, 100 and 200 mg/kg groups. The mice orally administration with CEE in three dosage, and the control and model mice were administration with the solvent of 0.5% CMC-Na for 7 days, respectively. At the last time drug administration, the mice were intratracheal instillation of 3 mg/kg LPS in saline (or with saline as a control) under anesthesia using sodium pentobarbital (40 mg/kg). After 3, 6 and 24 h of LPS stimulation, six mice were executed through injection of excessive sodium pentobarbital (80 mg/kg) at each time point. The blood and lung tissues were harvest for pathological detection.

### Histopathological analysis

The right lower lung was fixed with 4% polymethanol solution, then the lung tissue was dehydrated routinely, paraffin-embedded, sliced (thickness 5 μm), hematoxylin-eosin (H&E) stained, xylene transparent, neutral gum sealed to make paraffin-embedded pathological sections. Optical microscopy was used to observe whether the alveolar wall was thickened, hyperemia, inflammatory cell infiltration, interstitial inflammation, edema, alveolar necrosis and bleeding.

### Effect of CEE on cell numbers of the whole white blood and the classified cells

Blood samples were taken from eyeballs of mice in each group. Take 10 μL of blood samples from mice to EP tube containing 800 μL haemocyte dilution solution. The whole white blood and the classified cells were measured by automatic blood analyzer.

### Detection of wet/dry lung weight ratio, left lung dry weight/body weight ratio and lung index in lung injury mice

After eyeball bleeding, the mice were killed by cervical dislocation, then fixed on the operating table. After trachea exposure, the thoracic cavity was opened to observe the morphological changes of lungs. Then all the lungs were taken out and the wet weight of the whole lungs was weighed to calculate the lung index (lung index % = wet lung weight/body weight × 10^3^). After separating the left lung, weighing the wet weight, drying it in 60 °C blast drying box for 48 h to constant weight, taking out and precise fixed dry weight to calculate the wet/dry lung weight ratio (W/D) and the dry lung weight/body weight ratio of the left lung, which is mainly used to reflect the degree of pulmonary edema.

### Detection of pro-inflammation cytokine (TNF-*α*, IL-6) and anti-inflammatory cytokine (TGF-*β*1, IL-10) in lung tissue of mice by ELISA

Pre-cooled phosphate buffer saline (PBS) was used to prepare 10% lung tissue homogenate by electric homogenizer on ice. The expression levels of TNF-*α*, IL-6, TGF-*β*1 and IL-10 in lung tissue were detected by ELISA method according to the instruction of each kit from Wuhan Boster Biology Technology co. ltd. (Wuhan, China), respectively.

### Determination of the activity of T-AOC and the contents of MDA in lung tissue of mice

The lung tissue of mice was fully homogenized in an electric homogenizer with pre-cooled physiological saline, and 10% lung tissue homogenate was prepared according to the kit protocol from Nanjing Jiancheng Biotechnology Co., Ltd. (Nanjing, China).

### Statistical analysis

The present biological data were represented as mean ± SD. All statistical comparisons were calculated by means of a one-way ANOVA test followed by Dunett’s t-test with GraphPad Prism 6.0 statistical software. *P* < 0.05 and < 0.01 were regarded as statistically significant.

## Results

### Observation of mice symptoms

In control group, the mice were in good mental and developmental state, with normal feeding and drinking water, bright coat and normal feces. The mice in the model group had symptoms of reduced autonomic activity, mental depression, dyspnea, and accumulated with each other. At the same time, mucous substances were emitted from the mouth and nose, and severe diarrhea occurred. The intake of food and drinking water decreased significantly. Compared with the model group, the symptoms of CEE high dose group were significantly improved, while the symptoms of low dose group were slightly alleviated.

### Effect of CEE on the whole white cells and the clarified cells in lung injury mice

**Fig. 1:**
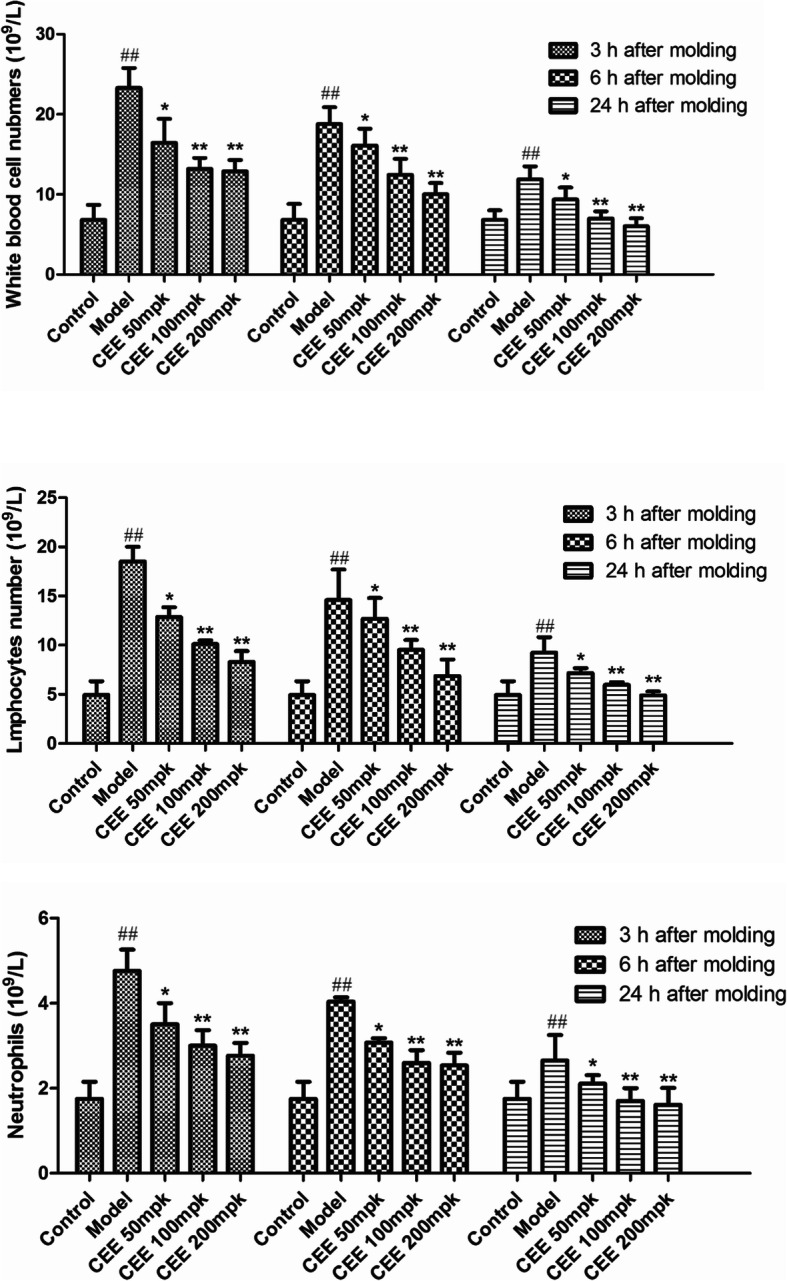
Effect of CEE on white blood cells in BALF of ALI mice (*n* = 18). ^##^*P* < 0.01 vs normal group, ^*^P < 0.05, ^**^P < 0.01 vs model group. CEE: *Chrysanthemum morifolium* Ramat. ethanol extract; ALI: acute lung injury

The increased numbers of white blood cells, lymphocytes and neutrophils related to the immunity and inflammation in the blood indicates that the body has an inflammatory response. Compared with the control group, the total number of the white blood cells, lymphocyte and neutrophils in the model group increased significantly at 3 h after modeling (Fig. 1, *P* < 0.01). Compared with model group, CEE in high, middle and low dose groups could significantly reduce the numbers of white blood cells, lymphocyte and neutrophils (Fig. 1, *P* < 0.05, *P* < 0.01). While at 6 h and 24 h after modeling, the number of white blood cells, lymphocyte and neutrophils was still significantly higher than that of control group. The three doses of CEE showed a significantly inhibition the inflammatory reaction in varying degrees (Fig. 1, *P* < 0.05, *P* < 0.01).

### Effect of CEE on lung index, left lung wet/dry weight ratio and left lung dry/body weight ratio in mice ALI

**Fig. 2:**
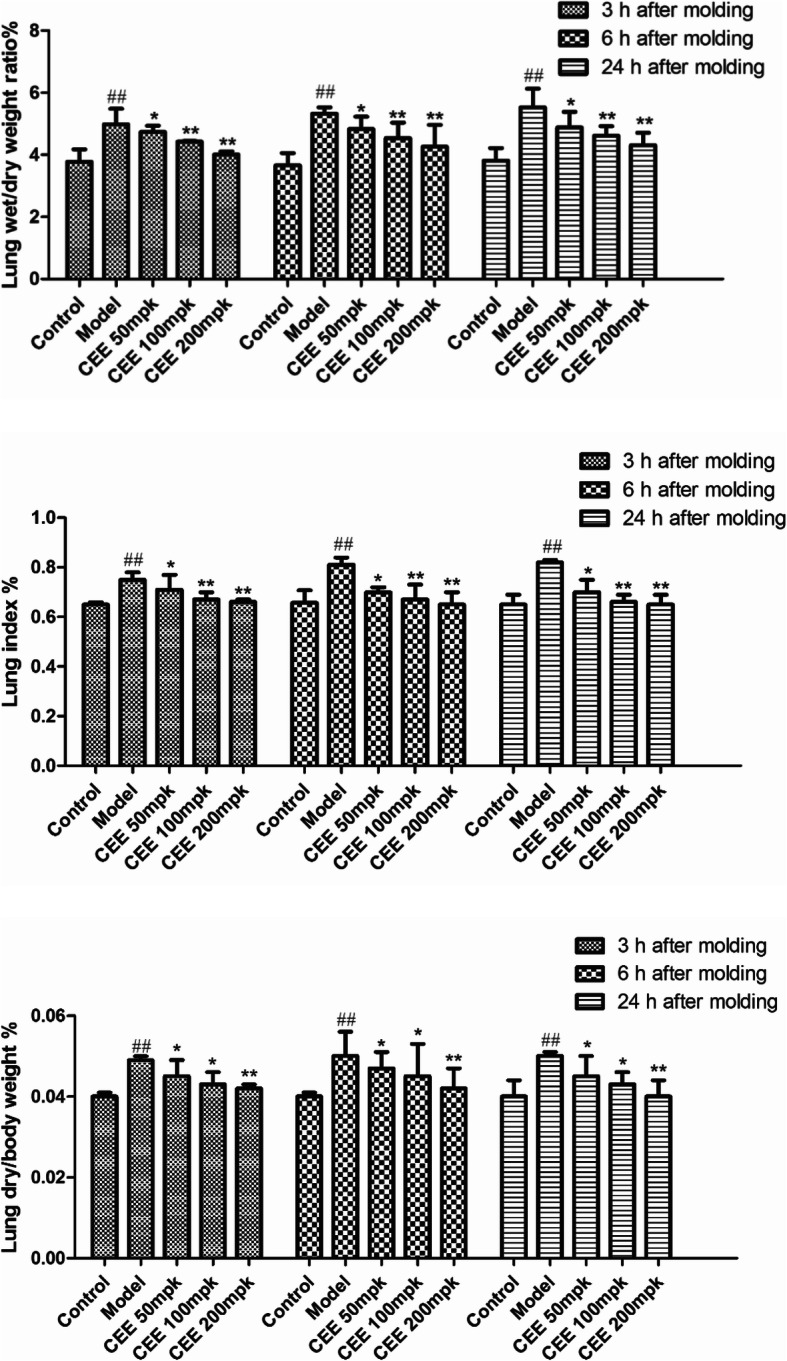
Effect of CEE on lung wet/dry weight ratio, lung index and lung dry/body weight ratio in ALI mice (n = 18). ^##^*P* < 0.01 vs normal group, ^*^P < 0.05, ^**^P < 0.01 vs model group. CEE: *Chrysanthemum morifolium* Ramat. ethanol extract; ALI: acute lung injury

Lung wet/dry weight ratio is used to evaluate the degree of lung edema. In Fig. 2, we can see that the lung index, lung wet/dry weight ratio and the lung dry/body weight ratio were significantly increased in each time point, compared with the control group (Fig. 2, *P* < 0.01). While all the three dosages of CEE could markedly reduce the lung index, the ratio of lung wet/dry weight and lung dry/body weight ratio at the time point of 3, 6 and 24 h, compared with the model group (Fig. 2, *P* < 0.05, *P* < 0.01), especially the high dose of CEE treated groups. This result reflected that CEE could alleviate lung edema in various degree.

### CEE improves lung histopathology in lung injury mice

**Fig. 3:**
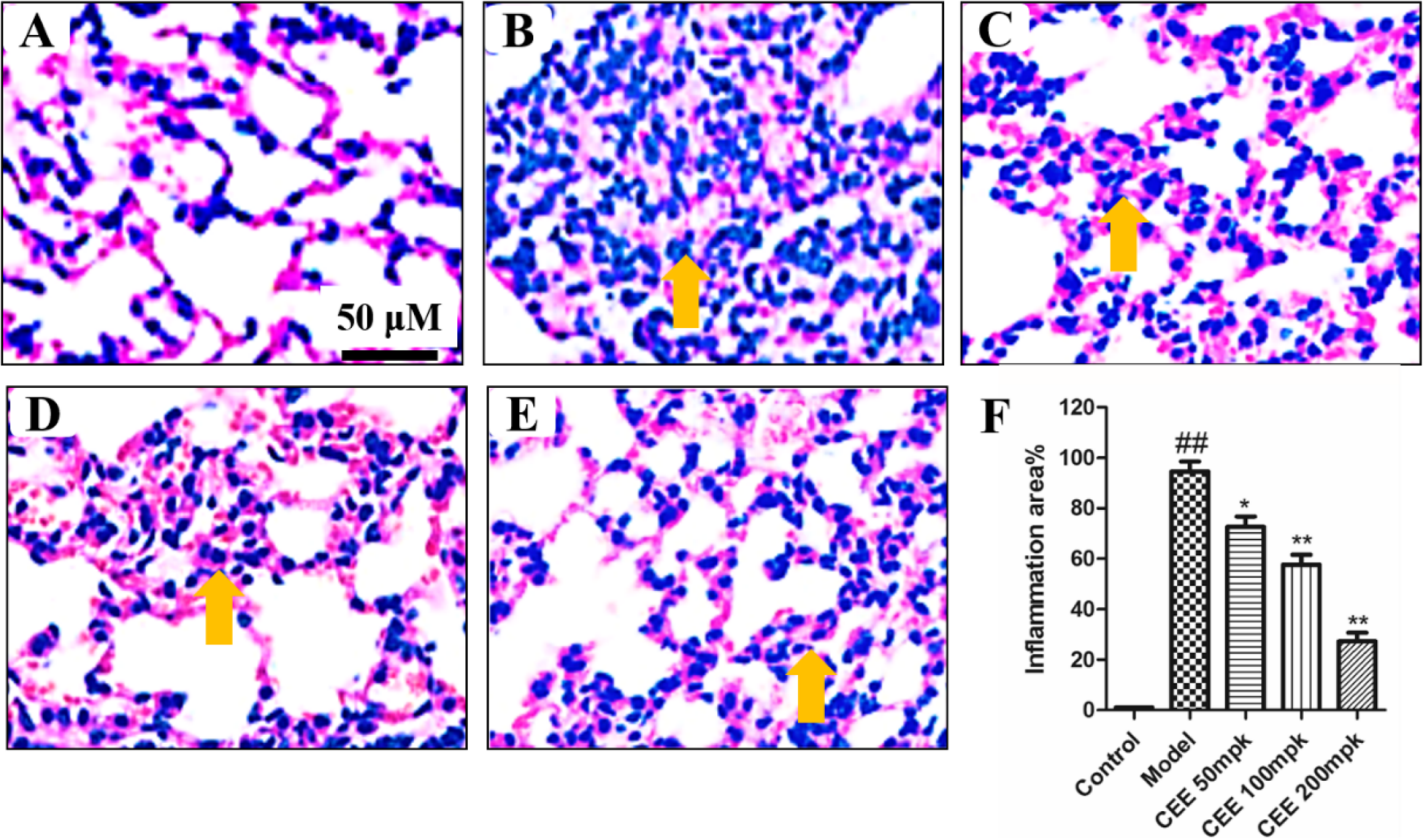
Effect of CEE on lung histopathology in ALI mice (H&E, Scale bar 50 μm). **a**. vehicle group, **b**. LPS model group, **c**. CEE 10 mg/kg, d. CEE 30 mg/kg **e**. CEE 60 mg/kg. **f**. the statistical data of the inflammation areas in each group. CEE: *Chrysanthemum morifolium* Ramat. ethanol extract; ALI, acute lung injury

Under the light microscopy of H&E section (Fig. 3), the alveolar cavity structure of control group was clear, alveolar wall was thin, and there was no obvious hyperemia and no neutrophil infiltration (Fig. 3a). In the model group, the lung tissue structure changed, alveolar wall was discontinuous, alveolar cavity disappeared in large area, alveolar wall became thicker, a large number of neutrophils and giant cells infiltrated the lungs, and there were obvious congestion and edema in the alveolar cavity, which also proved that the model of acute lung injury was successful (Fig. 3b). In CEE high, middle and low dose groups, the pathological changes were improved, the structure of the lung tissue was clear gradually, and the infiltration of pulmonary interstitium was improved significantly, especially in high dose group (Fig. 3c, d, e).

### Effect of CEE on the expression of TNF-*α*, IL-6, TGF-*β*1 and IL-10 in lung tissue of lung injury mice

Table 1 showed the levels of pro-inflammatory factor TNF-α and IL-6 in each groups.

**Table 1:** Effect of CEE on the levels of TNF-*α* and IL-6 in ALI mice

Group	Dose	3 h after molding	
		TNF-α (ng/g)	IL-6 (ng/g)
Normal	–	363.35 ± 41.80	60.06 ± 5.93
Model	–	539.5 ± 20.4^##^	101.31 ± 13.65^##^
CEE-low	50 mg/kg	482.75 ± 41.98^**^	95.62 ± 9.17^*^
CEE-medium	100 mg/kg	444.83 ± 12.11^**^	90.10 ± 5.11^**^
CEE-high	200 mg/kg	437.55 ± 12.12^**^	80.00 ± 8.74^**^
		**6 h after molding**	
Normal	–	363.42 ± 31.12	59.35 ± 5.47
Model	–	511.45 ± 31.9^##^	112.63 ± 5.01^##^
CEE-low	50 mg/kg	462.60 ± 18.11^*^	79.89 ± 10.12
CEE-medium	100 mg/kg	416.32 ± 19.62^*^	71.18 ± 4.76*
CEE-high	200 mg/kg	407.35 ± 4.99^**^	65.78 ± 9.20^**^
		**24 h after molding**	
Normal	–	362.49 ± 30.43	60.21 ± 4.86
Model	–	500.5 ± 20.4^##^	106.78 ± 9.13^##^
CEE-low	50 mg/kg	419.33 ± 8.92^**^	75.34 ± 13.53^*^
CEE-medium	100 mg/kg	392.00 ± 21.38^**^	65.39 ± 9.23^**^
CEE-high	200 mg/kg	380.05 ± 12.43^**^	62.41 ± 10.27^**^

Data are present as mean ± SD (n = 18). ^##^*P* < 0.01 vs normal group, ^*^P < 0.05, ^**^P < 0.01 vs model group. CEE: *Chrysanthemum morifolium* Ramat. ethanol extract; ALI: acute lung injury

After modeling 3 h, the levels of TNF-α and IL-6 in model group were significantly increased, compared with the control group (Table 1, *P* < 0.01), which reflect that the model was successfully established. At the 6 h after modeling, the levels of IL-6 still increasing, while TNF-α levels start decrease in model group, compared with the model group in 3 h. At the 24 h after modeling, the levels of TNF-α and IL-6 in model group were decreasing compared with the levels in 3 h. However, the levels of TNF-α and IL-6 in model group at 6 and 24 h still significantly higher than the control group (Table 1, *P* < 0.01). CEE treated groups were significantly reduced the levels of TNF-α and IL-6, compared with the model group (Table 1, *P* < 0.05, *P* < 0.01).

**Fig. 4:**
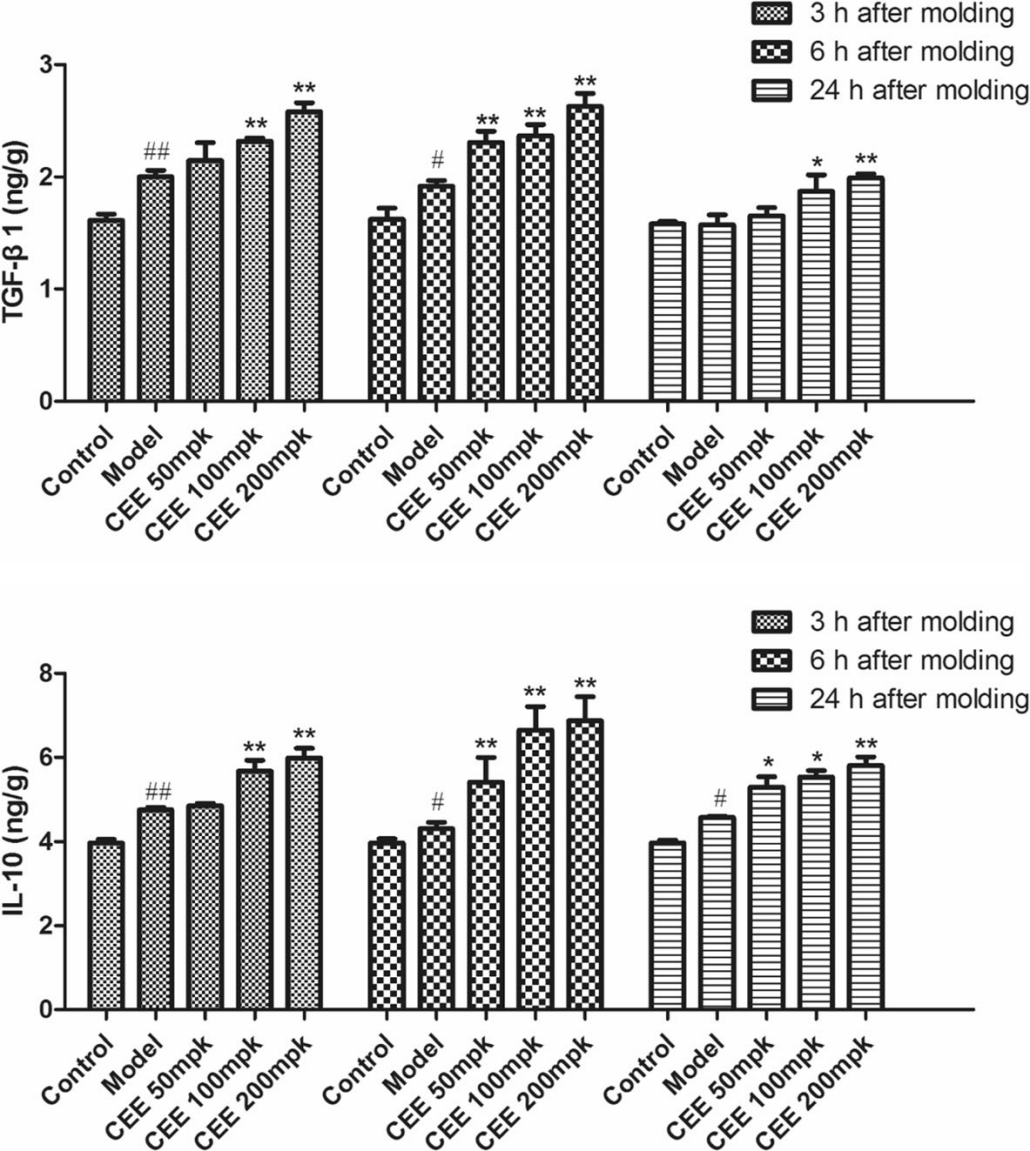
Effect of CEE on the levels of TGF-*β*1 and IL-10 in lung tissue of ALI mice (*n* = 18). ^##^*P* < 0.01 vs normal group, ^*^P < 0.05, ^**^P < 0.01 vs model group. CEE: *Chrysanthemum morifolium* Ramat. ethanol extract; ALI, acute lung injury

In addition, the levels of anti-inflammation factor TGF-*β*1 and IL-10 showed in Fig. 4. The result showed that at the time point of 3 and 6 h, the levels of TGF-*β*1 and IL-10 were significantly increased, compared with the control group (Fig. 4, *P* < 0.05, *P* < 0.01). CEE treated group could dramatically increase the levels of TGF-*β*1 and IL-10, compared with the model group (Fig. 4, *P* < 0.05, *P* < 0.01). While at 24 h, there was no obvious increase for the levels of TGF-*β*1 in model group, while CEE treated group still could increase the TGF-*β*1 levels, compared with model group, especially in the high dose of CEE (Fig. 4, *P* < 0.05, *P* < 0.01). Furthermore, IL-10 levels in model group at 24 h still higher than the control group (Fig. 4, *P* < 0.05). While the CEE treated groups also showed an increase in the levels of IL-10, compared with the model group (Fig. 4, *P* < 0.05, *P* < 0.01).

### Effect of CEE on the activity of T-AOC and MDA contents in lung tissue of lung injury mice

In Table 2, it can be seen that the MDA content in lung tissue of model group increased significantly at each time point, compared with control group (Table 2, *P* < 0.01). Compared with model group, the high, medium and low dosages of CEE groups could reduce the increase of MDA content in lung tissue (Table 2, *P* < 0.05, *P* < 0.01). At each time point, compared with the control group, the activity of T-AOC was significantly decreased in model group (Table 2, *P* < 0.01). Compared with model group, CEE treated groups could markedly increase T-AOC activity in lung tissue, especially the high dose of CEE treated group (Table 2, *P* < 0.05, *P* < 0.01). The result exhibited that CEE has an antioxidant effect in lung tissue for lung injury mice.

**Table 2:** Effect of CEE on the levels of T-AOC and MDA in ALI mice

Group	Dose	3 h after molding	
		T-AOC (U/mgprot)	MDA (nmol/mgprot)
Normal	–	2.78 ± 0.23	3.34 ± 0.39
Model	–	0.98 ± 0.06^##^	6.18 ± 0.31^##^
CEE-low	50 mg/kg	1.28 ± 0.08^*^	5.13 ± 0.03^*^
CEE-medium	100 mg/kg	1.97 ± 0.32^**^	4.65 ± 0.12^**^
CEE-high	200 mg/kg	2.05 ± 0.13^**^	4.26 ± 0.21^**^
		**6 h after molding**	
Normal	–	2.79 ± 0.22	3.35 ± 0.30
Model	–	0.98 ± 0.08^##^	6.23 ± 0.25^##^
CEE-low	50 mg/kg	1.27 ± 0.10^*^	5.15 ± 0.05^*^
CEE-medium	100 mg/kg	1.96 ± 0.42^**^	4.66 ± 0.33^**^
CEE-high	200 mg/kg	2.00 ± 0.08^**^	4.01 ± 0.53^**^
		**24 h after molding**	
Normal	–	2.79 ± 0.46	3.39 ± 0.19
Model	–	0.94 ± 0.02^##^	6.25 ± 0.45^##^
CEE-low	50 mg/kg	1.25 ± 0.05^*^	5.18 ± 0.09^*^
CEE-medium	100 mg/kg	2.07 ± 0.32^**^	4.68 ± 0.42^**^
CEE-high	200 mg/kg	2.58 ± 0.03^**^	4.00 ± 0.32^**^

Data are present as mean ± SD (n = 18). ^##^*P* < 0.01 vs normal group, ^*^P < 0.05, ^**^P < 0.01 vs model group. CEE: *Chrysanthemum morifolium* Ramat. ethanol extract; ALI: acute lung injury

### Bioactive component identification of CEE

**Fig. 5:**
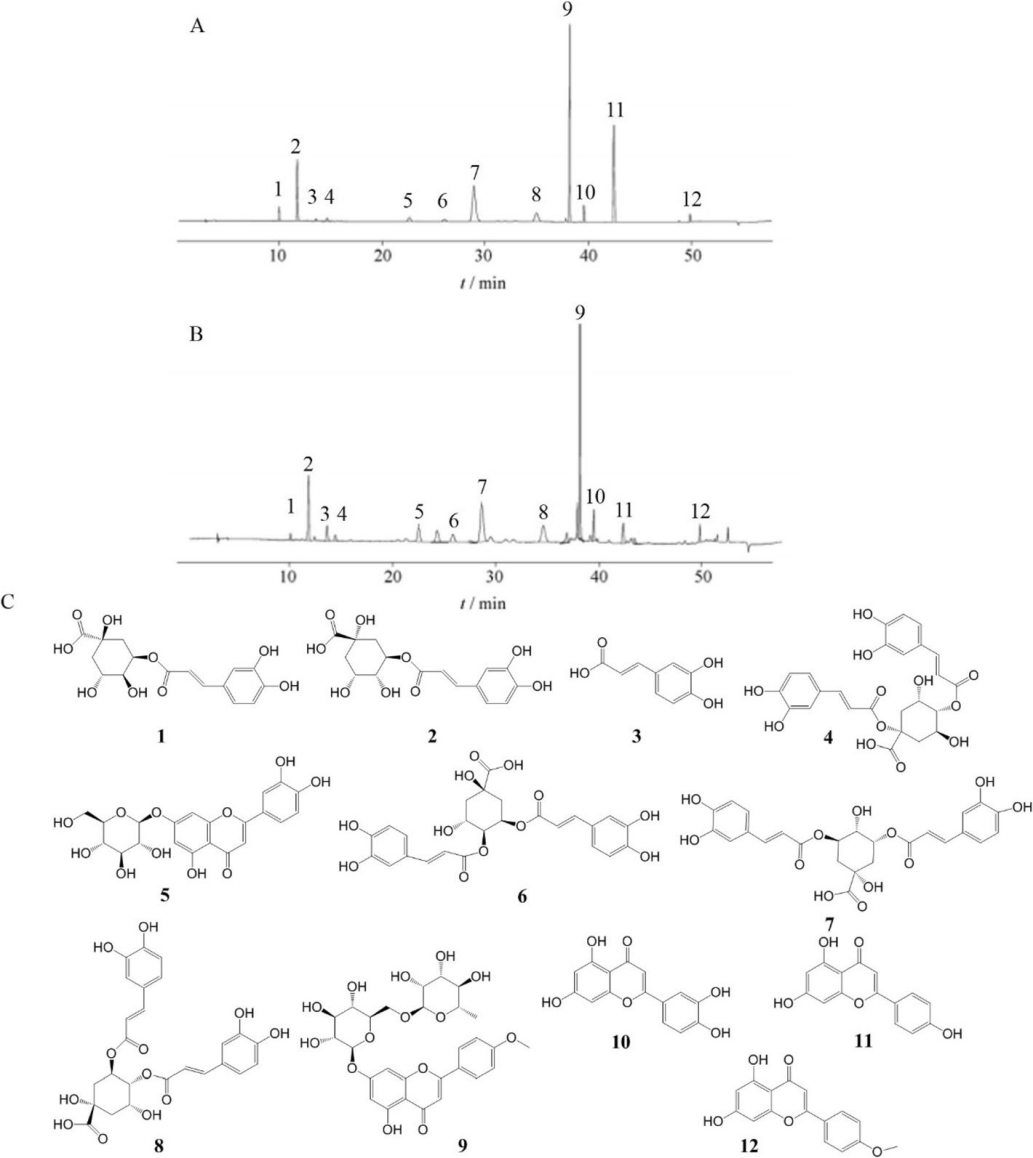
Bioactive constitutes of CEE. **a**. the HPLC chromatograph of the standard compounds 1–12. **b**. the HPLC chromatograph of CEE. **c**. the chemical structures of compounds 1–12: Neochlorogenic acid (1), chlorogenic acid (2), caffeic acid (3), 1,3-dicaffeoylquinic acids (4), cynaroside (5), isochlorogenic acid C (6), isochlorogenic acid A (7), isochlorogenic acid B (8), linarin (9), luteolin (10), apigenin (11) and acacetin (12)

Through the total ion chromatogram of CEE analysis, we have totally identified 12 components (Fig. 5) from CEE, they are neochlorogenic acid (1), chlorogenic acid (2), caffeic acid (3), 1,3-dicaffeoylquinic acids (4), cynaroside (5), isochlorogenic acid C (6), isochlorogenic acid A (7), isochlorogenic acid B (8), linarin (9), luteolin (10), apigenin (11), acacetin (12). We also compared the chromatograph peaks of the mixed standard compounds and the samples of CEE (Fig. 5a, b and c).

## Discussion

Endotoxin is a lipopolysaccharide (LPS) component of the outer membrane of Gram-negative bacilli [25]. It can cause alveolar epithelial cell injury and changes in lung structure and function, and enter the body to establish an ALI model after infection [26]. The direct consequence of ALI is alveolar edema, increased permeability, and the degree of microcirculation disorder in lung tissue, which can directly reflect the degree of lung injury [27]. Pulmonary microcirculation disturbance is mainly caused by structural changes and functional impairment of pulmonary vascular endothelial cells. Thus, the permeability of capillary is an index to judge pulmonary microcirculation disturbance. Lung wet/dry ratio, lung index and lung dry/body weight ratio of lung tissue can objectively reflect the relative degree of capillary permeability [27, 28]. In our study, the results showed that the water content in lung tissue of model mice increased significantly after LPS modeling, suggesting that the pulmonary capillary permeability increased significantly under acute lung injury. At three-time point, CEE (50, 100 and 200 mg/kg) could significantly reduce the lung wet/dry ratio, lung index and lung dry/body weight ratio. It can be concluded that CEE can reduce the permeability of pulmonary capillaries, improve pulmonary microcirculation, and then alleviate LPS-induced lung injury in ALI mice.

When the body stimulated by LPS, the activated macrophages will release a large number of pro-inflammatory cytokines such as TNF-*α*, IL-6 and IL-1*β*, which further induce neutrophils to migrate, aggregate in the interstitial and alveolar cavity, and produce respiratory bursts after the activation of aggregated neutrophils, leading to lung-lung eruption, and a series of lung tissue injuries, such as vascular congestion, pulmonary edema, alveolar atrophy, etc. [7, 29]. At the same time, compensatory anti-inflammatory response is also carried out, releasing endogenous anti-inflammatory factors such as soluble tumor necrosis factor receptor (sTNFR), interleukin-1 receptor antagonist (IL-1ra), IL-10 and TGF-*β*1 to achieve the balance of anti-inflammatory/inflammatory [27, 28, 30]. Therefore, whether drugs can inhibit inflammatory factors, increase anti-inflammatory factors, regulate the balance between inflammatory factors and anti-inflammatory factors in ALI, to achieve the goal of treating ALI. Therefore, in this study, we detected the levels of TNF-*α*, IL-6, TGF-*β*1 and IL-10 in lung tissue of mice. We observed that CEE can reduce the levels of TNF-*α* and IL-6 in lung tissue and increase the levels of TGF-*β*1 and IL-10. It is suggested that the protective effect of CEE on LPS-induced ALI is related to regulating the anti-inflammatory/inflammatory balance of ALI.

Furthermore, we also detected the oxidation and anti-oxidation activities in lung tissue of ALI mice. We found that CEE could also increase the activity of T-AOC, and reduce the lipid peroxidation products MDA contents, which reflect that CEE could also balance the oxidation/anti-oxidation in ALI mice. In addition, from the bioactive constitutes analysis, we found that the main chemical constitutes of CEE are flavonoids, phenolic acids and terpenoids.

## Conclusion

In conclusion, CEE showed a potential protective effect on lipopolysaccharide induced acute lung injury in mice, the protective mechanism of CEE may through the balance of the pro-inflammatory and anti-inflammatory factors, and the oxygen free radicals inhibition. While the bioactive constitues of CEE mainly focuses on flavonoids, phenolic acids and terpenoids.

## Data Availability

The datasets used and/or analyzed during the current study are available from the corresponding author on reasonable request.

## Abbreviations

CEE: *Chrysanthemum morifolium* Ramat. ethanol extract
LPS: Lipopolysaccharide
ALI: Acute lung injury
ATS: American Thoracic Society
ESICM: European Society of Critical Diseases
ARDS: Adult respiratory distress syndrome
TNF-*α*: Tumor necrosis factor-alpha
IL-6: Interleukin-6
TGF-*β*_1_: Transforming growth factor-beta 1
IL-10: Interleukin-10
H&E: Hematoxylin-eosin
CMC-Na: Cluster Methylcellulose Sodium
PBS: Phosphate buffer saline
DIC: Disseminated intravascular coagulation
sTNFR: soluble tumor necrosis factor receptor
IL-1ra: Interleukin-1 receptor antagonist
